# Effects of *CYP2B6 *G516T polymorphisms on plasma efavirenz and nevirapine levels when co-administered with rifampicin in HIV/TB co-infected Thai adults

**DOI:** 10.1186/1742-6405-7-8

**Published:** 2010-03-26

**Authors:** Sumonmal Uttayamakul, Sirirat Likanonsakul, Weerawat Manosuthi, Nuanjun Wichukchinda, Thareerat Kalambaheti, Emi E Nakayama, Tatsuo Shioda, Srisin Khusmith

**Affiliations:** 1 Department of Microbiology and Immunology, Faculty of Tropical Medicine, Mahidol University, Bangkok, Thailand; 2 Bamrasnaradura Infectious Diseases Institute, Department of Disease Control, Ministry of Public Health, Nonthaburi, Thailand; 3 National Institute of Health, Department of Medical Sciences, Ministry of Public Health, Nonthaburi, Thailand; 4 Research Institute of Microbial Disease, Osaka University, Osaka, Japan

## Abstract

**Background:**

Cytochrome P450 2B6 *(CYP2B6) *metabolizes efavirenz and nevirapine, the major core antiretroviral drugs for HIV in Thailand. Rifampicin, a critical component of tuberculosis (TB) therapy is a potent inducer of CYP enzyme activity. Polymorphisms of *CYP2B6 *and *CYP3A4 *are associated with altered activity of hepatic enzyme in the liver and pharmacokinetics resulting in treatment efficacy. This study aimed to investigate whether *CYP2B6 *or *CYP3A4 *polymorphisms had effects on plasma efavirenz and nevirapine concentrations when co-administered with rifampicin in HIV/TB co-infected Thai adults.

**Results:**

We studied 124 rifampicin recipients with concurrent HIV-1/TB coinfection, receiving efavirenz (600 mg/day) (n = 65) or nevirapine (400 mg/day) (n = 59) based antiretroviral therapy (ART). The frequencies of GG, GT and TT genotypes of *CYP2B6*-G516T were 38.46%, 47.69% and 13.85% in efavirenz group and 44.07%, 52.54% and 3.39% in nevirapine group, respectively. The mean 12-hour post-dose plasma efavirenz concentration in patients with TT genotype at weeks 6 and 12 of ART and 1 month after rifampicin discontinuation (10.97 ± 2.32, 13.62 ± 4.21 and 8.48 ± 1.30 mg/L, respectively) were significantly higher than those with GT (3.43 ± 0.29, 3.35 ± 0.27 and 3.21 ± 0.22 mg/L, respectively) (p < 0.0001) or GG genotypes (2.88 ± 0.33, 2.45 ± 0.26 and 2.08 ± 0.16 mg/L, respectively) (p < 0.0001). Likewise, the mean 12-hour post-dose plasma nevirapine concentration in patients carrying TT genotype at weeks 6 and 12 of ART and 1 month after rifampicin discontinuation (14.09 ± 9.49, 7.94 ± 2.76 and 9.44 ± 0.17 mg/L, respectively) tended to be higher than those carrying GT (5.65 ± 0.54, 5.58 ± 0.48 and 7.03 ± 0.64 mg/L, respectively) or GG genotypes (5.42 ± 0.48, 5.34 ± 0.50 and 6.43 ± 0.64 mg/L, respectively) (p = 0.003, p = 0.409 and p = 0.448, respectively). Compared with the effects of *CYP2B6-*516TT genotype, we could observe only small effects of rifampicin on plasma efavirenz and nevirapine levels. After 12 weeks of both drug regimens, there was a trend towards higher percentage of patients with *CYP2B6*-TT genotype who achieved HIV-1 RNA levels <50 copies/mL compared to those with GT or GG genotypes. This is the first report to demonstrate the effects of *CYP2B6 *G516T polymorphisms on plasma efavirenz and nevirapine concentrations when co-administered with rifampicin in HIV/TB co-infected Thai adults.

**Conclusions:**

*CYP2B6*-TT genotype had impact on plasma efavirenz and nevirapine concentrations, while rifampicin co-administration had only small effects.

## Background

Tuberculosis (TB) is the most common opportunistic infections in human immunodeficiency virus (HIV) infected individuals, accounting for more than 30% in Thailand, and up to 50% of them die during treatment [1]. The mortality is reduced in HIV-TB co-infected patients who have started the combination antiretroviral therapy after diagnosis of TB [2]. Concomitant administration of highly active antiretroviral therapy (HAART) and anti-TB medications is often complicated due to the drug-drug interaction and the adverse effect profile. Efavirenz and nevirapine based HAART regimens have mostly recommended to use as components of first-line antiretroviral drug regimens worldwide [3]. As efavirenz and nevirapine are potent non-nucleoside reverse transcriptase inhibitors (NNRTIs), they are the preferable option for initial antiretroviral treatments (ART) in HIV/TB co-infection. Rifampicin is a critical component of TB therapy while it is a potent inducer of cytochrome P450 (CYP) enzyme activity [4]. The available pharmacokinetic data showed that rifampicin reduced the plasma concentration of efavirenz and nevirapine of 13-25% and 40%, respectively [5-7]. Recently, efavirenz was shown *in vitro *to be primarily metabolized by hepatic CYP2B6, with minor contributions from CYP3A4 and CYP2A6 [4,8]. While rifampicin is an inducer of CYP3A4, nevirapine induces more CYP2B6 than CYP3A4 [9]. Nevirapine was also shown to be principally metabolized by CYP3A4 and CYP2B6 [10]. *CYP2B6 *and *CYP3A4 *genotypes are evidenced to be associated with altered activity of hepatic enzyme in the liver and pharmacokinetics that may influence efficacy of treatment, since rifampicin causes decrease in efavirenz and nevirapine concentrations [11-13].

The *CYP2B6 *and *CYP3A4 *genes are highly polymorphic [14] and are subject to pronounce interindividual variability in expression and activity. A single nucleotide polymorphism (SNP) at position 516 on the *CYP2B6 *gene has been widely reported to play an important role in the metabolism of antiretroviral drugs [15-18]. This *CYP2B6 *genetic variant affects the efavirenz and nevirapine pharmacokinetics [16,19,20] and associated with clinical response to nevirapine-containing regimens in children [16]. Significant advances have led to a greater understanding of interactions between genetic and host factors that influence the efficacy and toxicity of efavirenz [19,21]. However, the findings from one population may not be generalised to other populations due to the ethnic differences in drug effect and body weight of the patients. In Thailand, it has been recently reported that *CYP2B6*-G516T polymorphism significantly affected the drug metabolism of efavirenz in HIV-infected Thai children [22], while its impact on nevirapine concentrations was less pronounced after intra-partum single-dose nevirapine in HIV-infected mothers [23]. As efavirenz or nevirapine-based HAART is being used as the main therapy in Thailand, however, limited information was obtained so far among various Thai population regarding the influence of host genetic polymorphism on these drug levels especially nevirapine when co-administered with rifampicin which is essential for optimization of ARV dosage or drug-drug interaction. Therefore, the main objective of the present study is to investigate whether *CYP2B6 *and *CYP3A4 *polymorphisms could influence the plasma efavirenz and nevirapine levels when co-administered with rifampicin in HIV/TB infected Thai adults. The evaluation of clinical and immunological outcomes was also aimed.

## Methods

### Patients

One hundred and twenty four rifampicin recipients with concurrent HIV-1/TB coinfection were studied. Sixty-five of them received efavirenz (600 mg/day) based ART while 59 received nevirapine (400 mg/day) based ART. Initially, 142 patients were recruited for the study on a randomized control trial to compare the efficacy of efavirenz and nevirapine among HIV-infected patients receiving rifampicin at Bamrasnaradura Infectious Diseases Institute (BIDI), Nonthaburi since December 2006 [24]. They are ARV naïve with active tuberculosis and received rifampicin containing anti-TB regimens for 4-6 weeks prior to enrolment. The patients received oral lamivudine (150 mg) and stavudine (30 mg for those who weighed ≤ 60 kg and 40 mg for those who weighed >60 kg) every 12 hours. They were randomized to receive either efavirenz 600 mg at bedtime while fasting or nevirapine 200 mg every 12 hours after 2 weeks at a starting dose of 200 mg every 24 hours. The dosage of rifampicin was 450 mg/day for patients who weighed ≤ 50 kg and 600 mg/day for those who weighed >50 kg. The anti-TB drug regimen was isoniazid, rifampicin, ethambutol and pyrazinamide for the first two months, followed by isoniazid and rifampicin for the subsequent 4-7 months. Among 142 patients recruited, 25 patients (9 in the efavirenz group and 16 in the nevirapine group) failed to continue the study because of hepatitis (2 cases in the nevirapine group), skin rash (3 in the efavirenz group and 2 in the nevirapine group), death (2 in the efavirenz group and 6 in the nevirapine group), transfer to the other hospital (1 in the nevirapine group), or lost to follow up (4 in the efavirenz group and 5 in the nevirapine group). In the present study, we analyzed 124 patients who have a complete data set of plasma drug levels at week 6 and 12 of ART and 1 month after rifampicin discontinuation. The study was approved by Institutional Ethics Committees of Bamrasnaradura Infectious Diseases Institute and the Ministry of Public Health, Thailand and the written informed consents were obtained from all participants.

### Blood samples

EDTA bloods were collected from patients for SNP genotyping, CD4 T cell counts and HIV-1 viral load. Lithium heparinized bloods were collected after 12 hours of drug administration (C_12_) at weeks 6 and 12 of ART and after rifampicin discontinuation for 1 month for analysis of plasma efavirenz and nevirapine concentrations. The plasma were separated by centrifugation at 1800 g for 20 minutes and stored at -20°C.

### SNP genotyping of *CYP2B6 *and *CYP3A4*

The genomic DNA was extracted by using QIAamp DNA blood Mini kit (QIAGEN, Hilden, Germany) and stored at -20°C for SNP genotyping. Genotyping of allelic variants in *CYP2B6 *and *CYP3A4 *were carried out by real-time PCR using the allelic-specific fluorogenic 5' nuclease chain reaction assay by ABI PRISM 7500 sequence detection system (Applied Biosystems, Foster City, CA) as described previously [15]. Seven SNPs were genotyped: 4 SNPs of *CYP2B6*-G516T, -C777A, -A415G and -C1459T and 3 SNPs of *CYP3A4*-T566C, -T878C and C1088T. Each 25 μl PCR mixture contained 20 ng of genomic DNA, 900 nM primers, 200 nM TaqMan minor groove binder (MGB) probes and 12.5 μl TaqMan universal PCR master mix. The thermal cycler program was set up at 95°C for 10 minutes, and then repeated 40 cycles with 95°C for 15 seconds and 60°C for 1 minute. The plate was read by the allelic discrimination settings. The SNP assay was set up using SDS, version 1.3.0 as an absolute quantification assay. Post-assay analysis was done by using SDS software.

### Determination of plasma efavirenz and nevirapine concentration

Plasma efavirenz and nevirapine concentrations were measured by reverse phase high performance liquid chromatography (HPLC) method at the HIV-Netherlands-Australia-Thailand (HIV-NAT) Research Pharmacokinetic Laboratory, Chulalongkorn Medical Research Center (Bangkok, Thailand). HPLC was performed in accordance with the protocol developed by Department of Clinical Pharmacology, University Medical Center Nijmegan (Nijmegan, the Netherlands) [25].

### CD4 T lymphocyte counts and plasma HIV-1 RNA quantitation

The CD4 T lymphocyte counts were done at baseline and every 12 weeks after initiation of antiretroviral treatment by flow cytometry using monoclonal antibodies with three colors reagent (TriTEST, Becton Dickinson BioSciences, USA) and analyzed by FACScan flow cytometer (Becton Dickinson BioSciences, USA.). Plasma HIV-1 RNA was determined by RT-PCR at baseline and every 12 weeks after initiation of ART and quantified using the COBAS Amplicor, version 1.5 (Roche Diagnostics, USA). The lower detection limit for HIV-1 RNA level is 50 copies/mL.

### Statistical analysis

The different genotypes in relation to plasma drug levels were analysed by SPSS software version 14.0 (ID 5038562) (SPSS Inc., Chicago, IL, USA). If unpaired one-way analysis of variance (ANOVA) was significant (p < 0.05), then post hoc Scheffe's *F *test was applied for multiple comparison. When plasma drug levels of different time points were compared, paired T test was used. The CD4 T cell counts and HIV-1 viral load in patients carrying different genotypes were compared by Kruskal-Wallis test. A difference in proportion of patients who achieved plasma HIV-1 RNA < 50 copies/ml at week 12 of ART was evaluated by Chi square or Fisher's exact test. A *p *value of < 0.05 was considered statistically significant.

## Results

### Patient characteristics

The baseline characteristics of patients are shown in Table 1. All 124 patients were ethnically Thai and among these, 64.6% and 67.8% were male in efavirenz and nevirapine groups, respectively. The patients had the mean ages of 35.89 ± 8.17 and 38.03 ± 8.60 years and the mean body weights of 53.30 ± 9.79 and 54.39 ± 9.39 kg in efavirenz and nevirapine groups, respectively. Similar levels of laboratory parameters including alkaline phosphatase, aspartate aminotransferase, alanine aminotransferase, total bilirubin and direct bilirubin were seen in both patient groups. However, the levels of alkaline phosphatase among patients carrying TT genotype in efavirenz group were higher than those carrying GG or GT genotypes, but this difference was not statistically significant (p = 0.085). The median (interquartile range, IQR) CD4 T lymphocyte counts were similar in both groups. In nevirapine treatment group, the log number of plasma HIV-1 viral load among patients carrying GG, GT and TT genotypes seem to be significantly different (p = 0.041).

**Table 1:** Baseline characteristics of 124 HIV/TB co-infected patients with *CYP2B6*-G516T genotypes in efavirenz and nevirapine groups.

Baseline characteristics	Efavirenz group (n = 65)				Nevirapine group (n = 59)			
	*CYP2B6*-G516T				*CYP2B6*-G516T			
	
	GG	GT	TT	p-value	GG	GT	TT	p-value
	n = 25	n = 31	n = 9		n = 26	n = 31	n = 2	
Sex Male: Female	16: 9	21: 10	5: 4	0.795	17: 9	22: 9	1: 1	0.707
Age years, mean (SD)	36.48 (8.08)	35.68 (8.82)	35 (6.63)	0.882	36.48 (8.08)	35.68 (8.82)	35 (6.63)	0.467
Body weight kg, mean (SD)	52.9 (1.87)	53.94 (1.89)	52.22 (3.04)	0.872	54.62 (2.06)	54.7 (1.52)	46.5 (6.5)	0.489
Alkaline phosphatase, U/L, mean (SD)	149.2 (18.38)	137.1 (16.91)	233.9 (68.45)	0.085	150.25 (28.9)	113.97 (11.1)	125 (4)	0.458
Aspartate aminotransferase U/L, mean (SD)	32.8 (2.35)	40.48 (3.32)	43.22 (10.21)	0.202	48.54 (7.31)	35.58 (2.99)	26 (1)	0.167
Alanine aminotransferase, U/L, mean (SD)	27.0 (3.05)	28.55 (2.89)	31.22 (8.89)	0.821	29.81 (3.95)	27.94 (3.57)	23.5 (5.5)	0.877
Total bilirubin, mg/dL, mean (SD)	4.9 (4.34)	0.56 (0.55)	0.43 (0.07)	0.452	2.97 (2.4)	1.13 (0.57)	0.6 (0.1)	0.703
Direct bilirubin, mg/dL, mean (SD)	0.45 (0.14)	0.37 (0.12)	0.21 (0.05)	0.631	0.28 (0.047)	0.52 (0.199)	0.30 (0.1)	0.568
CD4 count, cells/μl, median (IQR)	41 (18-102)	54 (24-120)	67 (12.5-168)	0.818	35.5 (23.5-97)	45 (25-113)	30.5 (23)	0.595
Log Plasma HIV-1 viral load median (IQR)	5.90 (5.57-6.0)	5.93 (5.39-6.0)	5.64 (5.50-6.0)	0.729	5.86 (5.46-6.0)	5.60 (5.41-5.81)	5.80 (Q1 = 5.59)	0.041*

* Statistically significant by Kruskal-Wallis test. SD: standard deviation. IQR: interquartile range.

### Frequencies of *CYP2B6 *and *CYP3A4 *genetic polymorphisms

Seven SNPs: 4 SNPs of *CYP2B6*- G516T, -C777A, -A415G and -C1459T and 3 SNPs of *CYP3A4*-T566C, -T878C and -C1088T were genotyped. For *CYP2B6-*G516T, 38.46% (25/65) of GG genotype (wild-type), 47.69% (31/65) of GT genotype (heterozygous mutant) and 13.85% (9/65) of TT genotype (homozygous mutant) were found among patients in efavirenz group, while in nevirapine group, there were 44.07% (26/59) of *CYP2B6*-516GG genotype, 52.54% (31/59) of GT genotype and 3.39% (2/59) of TT genotype. The genotype frequencies of *CYP2B6*-C777A and -A415G in efavirenz and nevirapine groups were 100% of homozygous mutant AA and 100% of homozygous wild-type AA, respectively. For *CYP2B6*-C1459T, there were 98.5% (64/65) of CC homozygous wild-type and 1.5% (1/65) of CT heterozygous mutant in efavirenz group, and 91.5% (54/59) of CC homozygous wild-type, 6.8% (4/59) of CT heterozygous mutant and 1.7% (1/59) homozygous mutant in nevirapine group. Likewise, the genotype frequencies in *CYP3A4*-T566C and -C1088T were 100% of homozygous wild-type TT and homozygous mutant TT, respectively, in both efavirenz and nevirapine groups. For *CYP3A4*-T878C, there were 95.4% (62/65) of homozygous TT and 4.6% (3/65) of heterozygous TC, and 98.3% (58/59) of homozygous TT and 1.69% (1/59) of heterozygous TC in efavirenz and nevirapine groups, respectively.

### *CYP2B6-G516T *and *CYP3A4-T878C *genetic polymorphisms and plasma efavirenz and nevirapine concentrations

**Figure 1:**
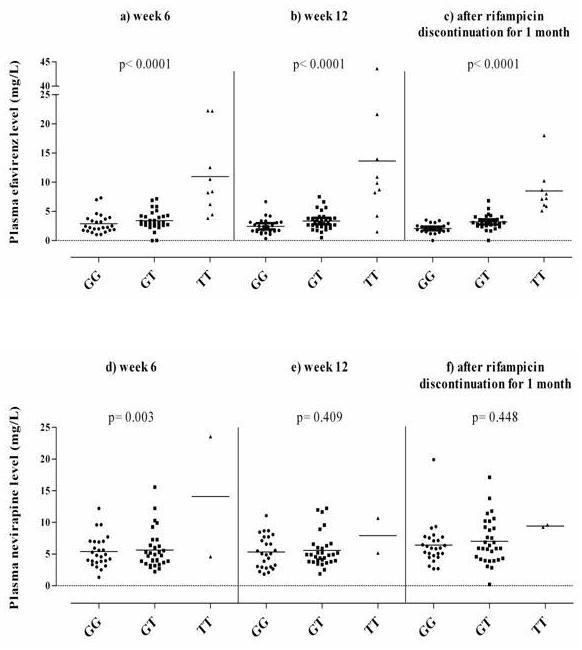
**Mean plasma efavirenz and nevirapine concentrations in HIV/TB adults with different genotypes of *CYP2B6*-G516T polymorphism**. The scatter diagram of plasma efavirenz (Fig.1a, b, c) and nevirapine distribution (Fig. 1d, e, f) at weeks 6 and 12 of ART and 1 month after rifampicin discontinuation. The numbers of GG, GT and TT genotype patients were 25, 31 and 9 in efavirenz group and 26, 31, 2 in nevirapine group.

Among 4 SNPs of *CYP2B6*-G516T, -C777A, -A415G and -C1459T being evaluated, the frequencies of wild-type, heterozygous mutant and homozygous mutant were well distributed only in *CYP2B6-*G516T polymorphism, therefore, the analysis of this gene polymorphism was further done in relation to plasma efavirenz and nevirapine levels. The mean plasma efavirenz concentration in patients with homozygous TT genotype at weeks 6 and 12 of ART and 1 month after rifampicin discontinuation (10.97 ± 2.32, 13.62 ± 4.21 mg/L and 8.48 ± 1.30 mg/L, respectively) were significantly higher than those with GT genotype (3.43 ± 0.29, 3.35 ± 0.27 mg/L and 3.21 ± 0.22 mg/L, respectively) or GG genotype (2.88 ± 0.33, 2.45 ± 0.26 and 2.08 ± 0.16 mg/L, respectively) (p < 0.0001) (Figure 1a, b, c). Similar results were found in nevirapine group (Figure 1d, e, f) in that the mean plasma drug concentration of patients with TT genotype at weeks 6 and 12 of ART and 1 month after rifampicin discontinuation (14.09 ± 9.49, 7.94 ± 2.76 and 9.44 ± 0.17 mg/L, respectively) tended to be higher than those with GT genotype (5.65 ± 0.54, 5.58 ± 0.48 and 7.03 ± 0.64 mg/L, respectively) or GG genotype (5.42 ± 0.48, 5.34 ± 0.50 and 6.43 ± 0.64 mg/L, respectively) (p = 0.003, p = 0.409 and p = 0.448, respectively).

One month after rifampicin discontinuation, there was a clear trend towards lower plasma efavirenz levels than those during concomitant rifampicin at week 6 and 12 of ART regardless of *CYP2B6 *G516T genotypes. In fact, when we evaluated effects of rifampicin on plasma efavirenz levels without stratifying *CYP2B6 *G516T polymorphisms, the plasma efavirenz levels after rifampicin discontinuation (3.5 ± 2.67 mg/L) were significantly lower than those at week 6 (4.26 ± 3.96 mg/L) (p = 0.043) and tended to be lower than those at week 12 (4.42 ± 5.97 mg/L) (p = 0.133). In contrast, plasma nevirapine levels at 1 month after rifampicin discontinuation (6.84 ± 3.4 mg/L) were significantly higher than those at week 6 (5.83 ± 3.6 mg/L, p = 0.034) and those at week 12 (5.56 ± 2.63 mg/L, p < 0.001). The reason for these discrepant results on effects of rifampicin on plasma efavirenz and nevirapine levels is not clear at present. Further studies including evaluation of plasma drug levels at time points other than 12-hour post-dose would be thus necessary. Nevertheless, we at least can conclude that the magnitude of effects on plasma efavirenz and nevirapine levels by rifampicin was much smaller than that by *CYP2B6 *516 TT genotype.

With respect to *CYP3A4*, the analysis was done in only *CYP3A4-*T878C, since there was no variation at the *CYP3A4*-T878C and -C1088T in our study subjects. The results showed that the mean plasma efavirenz concentration at weeks 6 and 12 of ART and 1 month after rifampicin discontinuation were 4.00 ± 0.42, 4.20 ± 0.72 and 3.48 ± 0.34 mg/L, respectively, in patients with homozygous TT genotype and 9.62 ± 6.35, 8.97 ± 6.33 and 3.87 ± 1.69 mg/L, respectively, in those with heterozygous TC genotype. Similarly, the mean plasma nevirapine concentration at weeks 6 and 12 of ART and 1 month after rifampicin discontinuation were 5.85 ± 0.48, 5.50 ± 0.34 and 6.80 ± 0.45 mg/L, respectively, in patients with homozygous TT genotype, and 4.8, 8.69 and 9.12 mg/L, respectively, in one patient with heterozygous mutant TC genotype. Although there was a trend towards higher plasma drug levels in patients with heterozygous mutant TC genotype, appropriate statistical evaluation of this difference was difficult due to small numbers of heterozygous mutant TC.

### CD4 T cell counts and HIV-1 viral load among patients with *CYP2B6-G516T *genotypes

**Figure 2:**
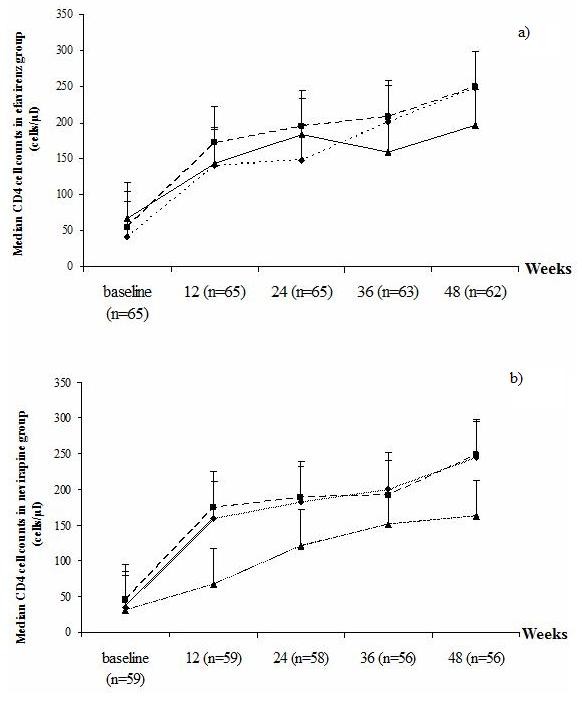
**Median CD4 T cell counts among HIV/TB adults with *CYP2B6*-G516T polymorphism at different time points**. (Black diamond) GG genotype, (Black square), GT genotype, (Black triangle) TT genotype in efavirenz (a) and nevirapine groups (b) at baseline, 12, 24, 36 and 48 weeks of ART.

The CD4 T cell counts among patients carrying different *CYP2B6 *genotypes in efavirenz and nevirapine groups are shown in Figure 2. The number of CD4 T cells in patients with TT, GT and GG genotypes increased in a similar manner at all time points at weeks 12, 24, 36 and 48 of ART compared to the baseline in both efavirenz and nevirapine groups. No significant difference in median CD4 T cell counts of each genotype at different time points was seen in efavirenz group (p = 0.818, 0.838, 0.783, 0.753 and 0.587 for baseline, weeks 12, 24, 36 and 48 of ART, respectively), whereas, in nevirapine group, the median CD4 T cell counts of patients with TT genotype seem to be lower than those with the other two genotypes at different time points, although this difference did not reach statistical significance (p = 0.595, 0.182, 0.554, 0.573 and 0.494, respectively) (Figure 2a, b).

As shown in Table 2, when the proportion of patients with HIV-1 RNA level <50 copies/mL (log 1.69) were compared among *CYP2B6*-G516T genotypes at week 12 of ART, 88.89% (8/9) of patients with TT genotype in efavirenz group could achieve the HIV-1 RNA levels <50 copies/mL, which were higher than those with GT genotype (77.42%, 24/31) and GG genotype (68%, 17/25), although this difference was not statistically significant (p = 0.430). Similarly, in nevirapine group, 100% (2/2) of those with TT genotype, 70.97% (23/31) of those with GT genotype and 60% (15/26) of those with GG genotype could achieve the HIV-1 RNA levels <50 copies/mL, but this difference also did not reach statistical significance (p = 0.288) due to small numbers of patients with homozygous TT genotype in this study. At weeks 24, 36 and 48 of ART, nearly all the patients achieved undetectable viral load, since viral load were not detected in 95.38% (62/65), 93.65% (59/63) and 87.9% (54/62), respectively, of efavirenz group and 96.55% (56/58), 94.64% (53/56) and 94.64% (53/56), respectively, of nevirapine group.

**Table 2:** Number of patients with plasma HIV-1 RNA < 50 copies/ml at week 12 of ART.

	Efavirenz group (N = 65)				Nevirapine group (N = 59)			
	*CYP2B6*-G516T				*CYP2B6*-G516T			
	
	GG n = 25	GT n = 31	TT n = 9	p-value*	GG n = 26	GT n = 31	TT n = 2	p-value**
No. of patients (%)	17 (68)	24 (77.42)	8 (88.89)	0.430	15 (60)	23 (70.97)	2 (100)	0.288

* Chi-square test

** Fisher's exact test

## Discussion

This is the first report to demonstrate the effects of *CYP2B6*-G516T and *CYP3A4-*T878C polymorphisms on plasma efavirenz and nevirapine concentrations in rifampicin-treated HIV/TB co-infected Thai adults. The results indicated that the wide interindividual variability of efavirenz concentrations is strongly influenced by *CYP2B6-*516TT genotype by the finding of significantly higher plasma efavirenz concentration at weeks 6 and 12 of ART and 1 month after rifampicin discontinuation compared to those with GT or GG genotype. Likewise, it seems to be that this *CYP2B6-*516TT would also influence nevirapine concentrations, although it was less pronounced probably due to the small samples size of homozygous mutant TT in our sample set. The present results were in line with the previous report on efavirenz pharmacokinetics when co-administration with rifampicin in HIV/TB co-infected Indian [26,27] and Ghana patients [28] in that plasma efavirenz was highest in patients with *CYP2B6*-516TT genotype when compared to those with GT or GG genotypes. While the heterozygous TC mutant in *CYP3A4-*T878C in this study seems to have some effects on plasma drug concentrations in patients at weeks 6 and 12 of ART and 1 month after rifampicin discontinuation in both efavirenz and nevirapine groups, further statistical analysis was not done due to the relatively less variation of *CYP3A4 *among Thai adults in this study. Further investigation should include a larger sample size with varying genotypes in order to draw a definite conclusion on the effect of *CYP3A4 *variations.

In this study, the frequency of *CYP2B6*-G516T among 124 Thai adults was 8.9%, which was close to that of our recent study on 237 HIV-infected Thai adults with different rate of CD4 T cell recovery after ARV treatment (9.7%) (submitted for publication) and slightly lower than what has been reported in Thai children (11%) [22]. Comparing to the other ethnic groups, it was higher than those of Japanese (3.3%) [15] and Caucasian (6%) [14], but lower than that of African-American (20%) [21] or African population (23%) [28,29]. Although the frequencies of *CYP2B6*-G516T were different among populations or ethnicity, the pharmacogenetic studies reported so far in HIV patients demonstrated that *CYP2B6 *516TT was definitely associated with plasma efavirenz concentration [15,19,21,29,30]. The findings of *CYP2B6 *516TT genotype in the present study support its effect on plasma efavirenz concentration in different ethnic group and gave additional information of this SNP on nevirapine based-ART when co-administered with rifampicin. The recent pharmacogenetic study in HIV patients co-administrated with efavirenz and rifampicin demonstrated that patients carrying TT genotype had significantly higher mean plasma efavirenz but lower oral clearance [28], indicating that rifampicin does not fully reverse the poor metabolizer phenotype and that TT genotype can be used to identify poor metabolizers of efavirenz even in patients co-administrated with rifampicin. Consistently, the present results also indicated that rifampicin coadministration in HIV/TB infected patients did not significantly alter plasma efavirenz and nevirapine levels in patients with TT genotype (p > 0.05). Other possible factors that might affect the plasma drug levels could be excluded since they were carefully controlled.

Although rifampicin can cause the decrease in NNRTI concentrations, the mean plasma efavirenz and nevirapine concentrations in all studied patients with TT, GT and GG genotypes had plasma drug levels above the minimum recommendation (1 mg/L for efavirenz and 3.4 mg/L for nevirapine). One important conclusion from our recent prospective and randomized clinical trial in patients with concurrent HIV/TB receiving rifampicin [24] is that the standard dosage of efavirenz 600 mg or nevirapine 400 mg per day and co-administration with rifampicin was adequate for HIV-1 suppression, however, variation in the plasma drug levels in some patients were found, which might be due to the genetic variations among individuals. Although we reported recently that high body weights of the patients were associated with a low efavirenz C_12 _at weeks 6 and 12 of ART [31], the present results demonstrated that the body weights did not differ among patients with different genotypes of *CYP2B6 *G516T polymorphism. The present results thus demonstrated that rifampicin has very small effects on efavirenz and nevirapine plasma drug. The advantage of our present study over previous studies is that plasma efavirenz and nevirapine concentrations during co-administration of rifampicin could be compared with those without rifampicin after completing TB drug treatment.

In general, the high plasma efavirenz and nevirapine levels could lead to the adverse effect such as rash, hepatitis, and neuropsychological toxicity [32,33]. In order to reduce such adverse effects, several studies attempted to test the feasibility of genotype-based dose reduction of efavirenz in African-American [34] and Japanese HIV infected patients [35] and showed that efavirenz dose reduction is feasible and can reduce efavirenz-associated central nervous system symptoms in homozygotes of *CYP2B6-*G516T. Although patients with *CYP2B6-*516TT in our cohort had obviously high plasma efavirenz levels at all time points and certain degree of central nervous system and psychiatric manifestations, they were all well tolerated with the adverse effects. The adverse drug events have not recorded in nevirapine based treatment probably due to the limited number of patients with homozygous TT. Since there were 7 cases who could not complete the study due to side effects [24] it is necessary to determine *CYP2B6 *G516T genotypes of these individuals in order to know whether *CYP2B6-*516TT homozygote in Thailand were all well tolerated with the adverse effects of efavirenz and nevirapine.

With respect to possible correlation of the variations in plasma efavirenz and nevirapine levels with the treatment outcome, our results indicated that the patients with *CYP2B6 *516TT genotype had a higher frequency of viral load suppression at week 12 of ART than those with GT and GG genotype. The CD4 T cell counts increased after treatment at all time points which were correlated with HIV-1 viral load reduction. When the effect of different *CYP2B6-*G516T genotypes was analysed, no difference was observed among patients with TT, GT and GG genotypes in both efavirenz and nevirapine groups. Collectively, it is indicated that the efavirenz and nevirapine-based ART co-administered with rifampicin are well correlated with virological and immunological outcomes in patients undergoing treatment for HIV and TB.

In summary, the *CYP2B6 *and *CYP3A4 *polymorphisms were analysed, for the first time, in HIV/TB co-infected Thai adults receiving efavirenz and nevirapine based-ART co-administered with rifampicin and the results indicated that only 516G>T in *CYP2B6 *gene, but not *CYP3A4 *gene polymorphism, gave the significant effects on plasma drug levels. Only small effects of rifampicin on efavirenz and nevirapine plasma concentration were observed. However, for further investigation, other SNPs such as *CYP2B6 *T983C or TGATC-*CYP2B6 *haplotypes which were shown to influence the NNRTI plasma drug levels [23,36,37] should be taken into account and larger sample size with varying genotypes should be included.

## Conclusions

*CYP2B6*-TT genotype had effects on both the plasma efavirenz and nevirapine concentrations in HIV/TB patients when co-administered with rifampicin. The information might be useful for better treatment of patients with HIV or HIV/TB.

## Competing interests

The authors declare that they have no competing interests.

## Authors' contributions

SU, SL, WM, NW, TK, SK participated in the study design. SU performed genotyping, CD4 counts and HIV-1 viral load determination, analysed the data and drafted the manuscript. EEN and NW took part in genotyping. SL and WM coordinated the study. TS and SK revised and finalised the manuscript. All authors read and approved the final manuscript.

## Author's information

SU is a Ph.D. candidate at the Faculty of Tropical Medicine, Mahidol University, Bangkok and deputy chief of Immunology and Virology Laboratory, Bamrasnaradura Infectious Diseases Institute (BIDI), Nonthaburi, Thailand. SL is a chief of Immunology and Virology Laboratory, BIDI. WM is a clinician who is taking care of HIV-1 infected patients and a principle investigator of a randomized control trial of efavirenz-based versus nevirapine-based antiretroviral therapy among HIV-infected patients receiving rifampicin. NW is the chief of Genetic Research Laboratory, National Institute of Health. TK is an assistant professor of Department of Microbiology and Immunology, Faculty of Tropical Medicine, Mahidol University. EEN is an assistant professor of Osaka University, Japan. TS is a professor of Osaka University and works on HIV-1 infection and host genome. SK is a professor of Department of Microbiology and Immunology, Faculty of Tropical Medicine, Mahidol University who does the research on malaria, TB and HIV and the supervisor of SU.

## References

[B1] Cain KP Anekthananon T Burapat C Akksilp S Mankhatitham W Srinak C Nateniyom S Sattayawuthipong W Tasaneeyapan T Varma JK Causes of death in HIV-infected persons who have tuberculosis, Thailand Emerg Infect Dis 2009 15 258 264 10.3201/eid1502.080942 19193270 PMC2657626

[B2] Manosuthi W Chottanapand S Thongyen S Chaovavanich A Sungkanuparph S Survival rate and risk factors of mortality among HIV/tuberculosis-coinfected patients with and without antiretroviral therapy J Acquir Immune Defic Syndr 2006 43 42 46 10.1097/01.qai.0000230521.86964.86 16885778

[B3] Hammer SM Saag MS Schechter M Montaner JS Schooley RT Jacobsen DM Thompson MA Carpenter CC Fischl MA Gazzard BG Gatell JM Hirsch MS Katzenstein DA Richman DD Vella S Yeni PG Volberding PA Treatment for adult HIV infection: 2006 recommendations of the International AIDS Society-USA panel JAMA 2006 296 827 843 10.1001/jama.296.7.827 16905788

[B4] Ward BA Gorski JC Jones DR Hall SD Flockhart DA Desta Z The cytochrome P450 2B6 (CYP2B6) is the main catalyst of efavirenz primary and secondary metabolism: implication for HIV/AIDS therapy and utility of efavirenz as a substrate marker of CYP2B6 catalytic activity J Pharmacol Exp Ther 2003 306 287 300 10.1124/jpet.103.049601 12676886

[B5] Lopez-Cortes LF Ruiz-Valderas R Viciana P Alarcon-Gonzalez A Gomez-Mateos J Leon-Jimenez E Sarasanacenta M Lopez-Pua Y Pachon J Pharmacokinetic interactions between efavirenz and rifampicin in HIV-infected patients with tuberculosis Clin Pharmacokinet 2002 41 681 690 10.2165/00003088-200241090-00004 12126459

[B6] Patel A Patel K Patel J Shah N Patel B Rani S Safety and antiretroviral effectiveness of concomitant use of rifampicin and efavirenz for antiretroviral-naive patients in India who are coinfected with tuberculosis and HIV-1 J Acquir Immune Defic Syndr 2004 37 1166 1169 10.1097/01.qai.0000135956.96166.f0 15319677

[B7] Ribera E Pou L Lopez RM Crespo M Falco V Ocana I Ruiz I Pahissa A Pharmacokinetic interaction between nevirapine and rifampicin in HIV-infected patients with tuberculosis J Acquir Immune Defic Syndr 2001 28 450 453 11744833 10.1097/00042560-200112150-00007

[B8] Desta Z Saussele T Ward B Blievernicht J Li L Klein K Flockhart DA Zanger UM Impact of CYP2B6 polymorphism on hepatic efavirenz metabolism in vitro Pharmacogenomics 2007 8 547 558 10.2217/14622416.8.6.547 17559344

[B9] Pinzani V Faucherre V Peyriere H Blayac JP Methadone withdrawal symptoms with nevirapine and efavirenz Ann Pharmacother 2000 34 405 407 10.1345/aph.19134 10917395

[B10] Erickson DA Mather G Trager WF Levy RH Keirns JJ Characterization of the in vitro biotransformation of the HIV-1 reverse transcriptase inhibitor nevirapine by human hepatic cytochromes P-450 Drug Metab Dispos 1999 27 1488 1495 10570031

[B11] Cohen K Grant A Dandara C McIlleron H Pemba L Fielding K Charalombous S Churchyard G Smith P Maartens G Effect of rifampicin-based antitubercular therapy and the cytochrome P450 2B6 516G>T polymorphism on efavirenz concentrations in adults in South Africa Antivir Ther 2009 14 687 695 19704172 PMC3837290

[B12] Cabrera SE Santos D Valverde MP Dominguez-Gil A Gonzalez F Luna G Garcia MJ Influence of the cytochrome P450 2B6 genotype on population pharmacokinetics of efavirenz in human immunodeficiency virus patients Antimicrob Agents Chemother 2009 53 2791 2798 10.1128/AAC.01537-08 19433561 PMC2704695

[B13] Cohen K van Cutsem G Boulle A McIlleron H Goemaere E Smith PJ Maartens G Effect of rifampicin-based antitubercular therapy on nevirapine plasma concentrations in South African adults with HIV-associated tuberculosis J Antimicrob Chemother 2008 61 389 393 10.1093/jac/dkm484 18096560

[B14] Lang T Klein K Fischer J Nussler AK Neuhaus P Hofmann U Eichelbaum M Schwab M Zanger UM Extensive genetic polymorphism in the human CYP2B6 gene with impact on expression and function in human liver Pharmacogenetics 2001 11 399 415 10.1097/00008571-200107000-00004 11470993

[B15] Tsuchiya K Gatanaga H Tachikawa N Teruya K Kikuchi Y Yoshino M Kuwahara T Shirasaka T Kimura S Oka S Homozygous CYP2B6 *6 (Q172H and K262R) correlates with high plasma efavirenz concentrations in HIV-1 patients treated with standard efavirenz-containing regimens Biochem Biophys Res Commun 2004 319 1322 1326 10.1016/j.bbrc.2004.05.116 15194512

[B16] Saitoh A Sarles E Capparelli E Aweeka F Kovacs A Burchett SK Wiznia A Nachman S Fenton T Spector SA CYP2B6 genetic variants are associated with nevirapine pharmacokinetics and clinical response in HIV-1-infected children AIDS 2007 21 2191 2199 10.1097/QAD.0b013e3282ef9695 18090046

[B17] Powers V Ward J Gompels M CYP2B6 G516T genotyping in a UK cohort of HIV-positive patients: polymorphism frequency and influence on efavirenz discontinuation HIV Med 2009 10 520 523 10.1111/j.1468-1293.2009.00718.x 19486190

[B18] Haas DW Gebretsadik T Mayo G Menon UN Acosta EP Shintani A Floyd M Stein CM Wilkinson GR Associations between CYP2B6 polymorphisms and pharmacokinetics after a single dose of nevirapine or efavirenz in African americans J Infect Dis 2009 199 872 880 10.1086/597125 19239339 PMC2784690

[B19] King J Aberg JA Clinical impact of patient population differences and genomic variation in efavirenz therapy AIDS 2008 22 1709 1717 10.1097/QAD.0b013e32830163ad 18753940

[B20] Saitoh A Fletcher CV Brundage R Alvero C Fenton T Hsia K Spector SA Efavirenz pharmacokinetics in HIV-1-infected children are associated with CYP2B6-G516T polymorphism J Acquir Immune Defic Syndr 2007 45 280 285 17356468 10.1097/QAI.0b013e318040b29e

[B21] Haas DW Ribaudo HJ Kim RB Tierney C Wilkinson GR Gulick RM Clifford DB Hulgan T Marzolini C Acosta EP Pharmacogenetics of efavirenz and central nervous system side effects: an Adult AIDS Clinical Trials Group study AIDS 2004 18 2391 2400 15622315

[B22] Puthanakit T Tanpaiboon P Aurpibul L Cressey TR Sirisanthana V Plasma efavirenz concentrations and the association with CYP2B6-516G>T polymorphism in HIV-infected Thai children Antivir Ther 2009 14 315 320 19474465

[B23] Chantarangsu S Cressey TR Mahasirimongkol S Capparelli E Tawon Y Ngo-Giang-Huong N Jourdain G Lallemant M Chantratita W Influence of CYP2B6 polymorphisms on the persistence of plasma nevirapine concentrations following a single intra-partum dose for the prevention of mother to child transmission in HIV-infected Thai women J Antimicrob Chemother 2009 19812066 10.1093/jac/dkp351 PMC2775665

[B24] Manosuthi W Sungkanuparph S Tantanathip P Lueangniyomkul A Mankatitham W Prasithsirskul W Burapatarawong S Thongyen S Likanonsakul S Thawornwa U Prommool V Ruxrungtham K A randomized trial comparing plasma drug concentrations and efficacies between 2 nonnucleoside reverse-transcriptase inhibitor-based regimens in HIV-infected patients receiving rifampicin: the N2R Study Clin Infect Dis 2009 48 1752 1759 10.1086/599114 19438397

[B25] Hollanders RM van Ewijk-Beneken Kolmer EW Burger DM Wuis EW Koopmans PP Hekster YA Determination of nevirapine, an HIV-1 non-nucleoside reverse transcriptase inhibitor, in human plasma by reversed-phase high-performance liquid chromatography J Chromatogr B Biomed Sci Appl 2000 744 65 71 10.1016/S0378-4347(00)00231-0 10985567

[B26] Ramachandran G Hemanth Kumar AK Rajasekaran S Kumar P Ramesh K Anitha S Narendran G Menon P Gomathi C Swaminathan S CYP2B6 G516T polymorphism but not rifampin coadministration influences steady-state pharmacokinetics of efavirenz in human immunodeficiency virus-infected patients in South India Antimicrob Agents Chemother 2009 53 863 868 10.1128/AAC.00899-08 19124658 PMC2650539

[B27] Ramachandran G Ramesh K Hemanth Kumar AK Jagan I Vasantha M Padmapriyadarsini C Narendran G Rajasekaran S Swaminathan S Association of high T allele frequency of CYP2B6 G516T polymorphism among ethnic south Indian HIV-infected patients with elevated plasma efavirenz and nevirapine J Antimicrob Chemother 2009 63 841 843 10.1093/jac/dkp033 19218571

[B28] Kwara A Lartey M Sagoe KW Xexemeku F Kenu E Oliver-Commey J Boima V Sagoe A Boamah I Greenblatt DJ Court MH Pharmacokinetics of efavirenz when co-administered with rifampin in TB/HIV co-infected patients: pharmacogenetic effect of CYP2B6 variation J Clin Pharmacol 2008 48 1032 1040 10.1177/0091270008321790 18728241 PMC2679896

[B29] Wang J Sonnerborg A Rane A Josephson F Lundgren S Stahle L Ingelman-Sundberg M Identification of a novel specific CYP2B6 allele in Africans causing impaired metabolism of the HIV drug efavirenz Pharmacogenet Genomics 2006 16 191 198 10.1097/01.fpc.0000230119.34205.8a 16495778

[B30] Rotger M Tegude H Colombo S Cavassini M Furrer H Decosterd L Blievernicht J Saussele T Gunthard HF Schwab M Eichelbaum M Telenti A Zanger UM Predictive value of known and novel alleles of CYP2B6 for efavirenz plasma concentrations in HIV-infected individuals Clin Pharmacol Ther 2007 81 557 566 10.1038/sj.clpt.6100072 17235330

[B31] Manosuthi W Sungkanuparph S Tantanathip P Mankatitham W Lueangniyomkul A Thongyen S Eampokarap B Uttayamakul S Suwanvattana P Kaewsaard S Ruxrungtham K Body weight cutoff for daily dosage of efavirenz and 60-week efficacy of efavirenz-based regimen in human immunodeficiency virus and tuberculosis coinfected patients receiving rifampin Antimicrob Agents Chemother 2009 53 4545 4548 10.1128/AAC.00492-09 19667281 PMC2764182

[B32] Kappelhoff BS van Leth F Robinson PA MacGregor TR Baraldi E Montella F Uip DE Thompson MA Russell DB Lange JM Beijnen JH Huitema AD Are adverse events of nevirapine and efavirenz related to plasma concentrations? Antivir Ther 2005 10 489 498 16038474

[B33] Ananworanich J Moor Z Siangphoe U Chan J Cardiello P Duncombe C Phanuphak P Ruxrungtham K Lange J Cooper DA Incidence and risk factors for rash in Thai patients randomized to regimens with nevirapine, efavirenz or both drugs AIDS 2005 19 185 192 10.1097/00002030-200501280-00011 15668544

[B34] Torno MS Witt MD Saitoh A Fletcher CV Successful use of reduced-dose efavirenz in a patient with human immunodeficiency virus infection: case report and review of the literature Pharmacotherapy 2008 28 782 787 10.1592/phco.28.6.782 18503405

[B35] Gatanaga H Hayashida T Tsuchiya K Yoshino M Kuwahara T Tsukada H Fujimoto K Sato I Ueda M Horiba M Hamaguchi M Yamamoto M Takata N Kimura A Koike T Gejyo F Matsushita S Shirasaka T Kimura S Oka S Successful efavirenz dose reduction in HIV type 1-infected individuals with cytochrome P450 2B6 *6 and *26 Clin Infect Dis 2007 45 1230 1237 10.1086/522175 17918089

[B36] Kwara A Lartey M Sagoe KW Kenu E Court MH CYP2B6, CYP2A6 and UGT2B7 genetic polymorphisms are predictors of efavirenz mid-dose concentration in HIV-infected patients AIDS 2009 23 2101 2106 10.1097/QAD.0b013e3283319908 19779319 PMC2875867

[B37] Wyen C Hendra H Vogel M Hoffmann C Knechten H Brockmeyer NH Bogner JR Rockstroh J Esser S Jaeger H Harrer T Mauss S van Lunzen J Skoetz N Jetter A Groneuer C Fatkenheuer G Khoo SH Egan D Back DJ Owen A Impact of CYP2B6 983T>C polymorphism on non-nucleoside reverse transcriptase inhibitor plasma concentrations in HIV-infected patients J Antimicrob Chemother 2008 61 914 918 10.1093/jac/dkn029 18281305 PMC3596857

